# Wild Yeasts as Reservoirs of Bacterial Diversity: Biotechnological Insights from 16S rRNA Metabarcoding

**DOI:** 10.3390/foods15020262

**Published:** 2026-01-11

**Authors:** Eugenia Iturritxa, Nebai Mesanza, María-Jesús Torija

**Affiliations:** 1 Department of Forest Science, Neiker-BRTA, Instituto Vasco de Investigación y Desarrollo Agrario, Granja Modelo s/n, Antigua Carretera Nacional 1, Km. 355, 01192 Arkaute, Spain; nmesanza@neiker.eus; 2 Grup de Recerca Biotecnologia Enológica, Department de Bioquímica i Biotecnologia, Facultat d’Enologia, Universitat Rovira i Virgili, C/Marcel·lí Domingo 1, 43007 Tarragona, Spain

**Keywords:** axenic yeast cultures, fermentation, *Vitis*, *Quercus*, endobacteria, yeast

## Abstract

Recently acquired evidence indicates that bacteria can utilise yeasts as survival niches. This study investigated the presence of hidden, intracellular bacteria (endobacteria) within wild yeasts collected from natural ecosystems and evaluated whether biotechnological processes influenced these bacterial communities. We examined the microbiotas of 28 axenic cultures of wild yeasts; these were selected due to their potential brewing and biocontrol uses and were isolated from habitats associated with *Quercus* and *Vitis*. We also analysed the microbiotas present after these strains were used to ferment beer wort. Bacterial communities were characterised using 16S rRNA gene amplicon metagenomics. The results indicate that yeast strains and their endobacterial partners have coevolved, and their compositions are shaped by the environmental conditions. Substantial bacterial diversity was detected across strains in both axenic cultures and post-fermentation samples. The ecological origin of the yeast (oak- or grape-associated) did not significantly affect the endobacterial community structure. Across all samples, the dominant phyla were Proteobacteria, Actinobacteria, Firmicutes, and Cyanobacteria, with Proteobacteria representing over 90% of sequences. Most bacterial genera were shared between axenic and fermentation conditions. However, *Escherichia* and *Comamonas* predominated in axenic cultures, while *Parvibaculum* dominated after fermentation. These findings suggest that yeasts constitute stable microhabitats for bacterial communities, and their relative abundances can shift during fermentation processes.

## 1. Introduction

Eukaryotes have experienced diversification owing to the persistent colonisation of beneficial microorganisms [1]. Bacterial interactions between plants and animals have been studied for many years. Additionally, fungi and bacteria coexist in various natural communities, where their interactions and mutual influences on one another’s metabolism and on ecosystems have been documented [2,3,4]. However, the study of bacterial interactions with yeasts in biotechnological processes offers a novel microbiological perspective. Yeast endobacteria play different roles, ranging from mutualism to antagonism, and may confer certain benefits to their associated yeast hosts. Thus, they show potential to facilitate biotechnological processes, where they could provide the cellular machinery for genetic expression [5,6]. Ponomarova et al. [7] reported the de novo assembly of a stable community between yeasts and commensalistic lactic acid bacteria, in which the yeast allowed the growth of lactic acid bacteria through endogenous multicomponent cross-feeding.

The intracellular establishment of prokaryotes within eukaryotic cells—with the latter providing essential elements for balanced, safe, and productive maintenance—suggests that bacteria are incorporated into eukaryotic cells via ingestion as food [8,9] or by inducing their internalisation to cope with environmental conditions [10,11]. The yeast vacuole is an organelle that specialises in food digestion and autophagy [12,13]; it is a nutrient storage site and a niche for intracellular bacteria [13,14]. Therefore, within the vacuole, endobacteria are protected against environmental stress and nutrient availability [11,15,16]. Additionally, endosymbiotic bacteria enhance yeasts’ digestive capacities, tolerances, and stress adaptation [17]. Although many studies have identified these endosymbiotic bacteria using culture-independent methods [18,19,20,21], cultivation remains the most accurate method of characterising these intracellular bacteria. However, in many cases, this is not possible due to a lack of knowledge regarding the types of culture media and environmental conditions required for their isolation and growth [17].

Symbiosis is widespread in fermentation and biological control processes. For example, in fermented foods such as yoghurt, milk, kefir, and sourdough, various microorganisms are present, and they participate in these foods’ production through different associations [22,23,24,25]. In biological control, combinations of two or more microbial strains are used to improve the stability and efficiency of disease inhibition [26,27]. The biotechnological potential of microbial consortia is promising, with possible applications in biopolymers, bioenergy, biochemicals, bioremediation, biocontrol, and food [28].

In this study, we investigated the cryptic bacterial communities associated with axenic cultures of wild yeasts, examining how community structure is influenced by the yeast’s ecological origin [29,30] and how it changes during fermentation. Using a novel detection and characterisation approach based on 16S rRNA gene amplicon sequencing, we analysed bacterial communities associated with 28 axenic yeast isolates from vineyard and forest ecosystems, comparing them with the communities present after wort fermentation. Our findings reveal that traditional yeast isolation methods may overlook resident bacterial consortia, uncovering previously unrecognised yeast–bacterium associations that could impact fermentation performance, flavour development, product stability, and process robustness. These results provide a basis for enhancing the control and optimisation of biotechnological processes involving complex microbial consortia.

## 2. Materials and Methods

### 2.1. Yeast Strains and Conditions

In this study, 28 wild yeasts were used: 13 isolated from *Quercus* (25q, 29q, 49q, 53q, 70q, 86q, 90q, 104q, 118q, 138q, 142q, 146q, and 163q) and 15 from Rioja vineyards (2g, 4g, 5g, 7g, 8g, 9g, 10g, 11g, 12g, 13g, 14g, 15g, 16g, 17g, and 20g) in the Basque Country (Spain). These strains belonged to eight yeast species: *Hanseniaspora uvarum* (11g); *Lachancea thermotolerans* (104q, 118q, 163q, 12g, 13g, 14g, 15g, and 8g); *Metschnikowia pulcherrima* (9g); *Naganishia albida* (5g); *Pichia kudriavzevii* (138q, 142q, 146q, and 7g); *Saccharomyces paradoxus* (20g, 25q, 29q, 49q, 53q, 70q, 86q, and 90q); *Starmerella bacillaris* (10g and 4g); and *Torulaspora delbrueckii* (16g, 17g, and 2g), Detailed information on the origin of the isolates analysed in this study is provided in Supplementary Table S1. The strains were selected based on their enzymatic profiles and the fact that they exhibited optimal properties for brewing and biological control [29,30].

Two sets of samples were analysed: F0, comprising axenic cultures of the 28 selected wild yeasts, and F1, comprising the same yeasts after being used to ferment a low-density beer wort (density 1025 g/L and pH 5.67) prepared with a European Lager (DIY Beer Coopers, Regency Park, Australia) [29]. Fermentation procedures were conducted in 250 mL Erlenmeyer flasks containing 150 mL of wort, at 20 °C and without stirring; each flask was sealed with a rubber stopper and fitted with an airlock. F1 samples were collected after 7 days of fermentation.

Axenic cultures of the selected yeasts were grown in a yeast peptone dextrose (YPD) medium (10 g/L yeast extract, 20 g/L peptone, 20 g/L dextrose, and 15 g/L bacteriological agar), which was supplemented with chloramphenicol (100 μg/mL) to prevent bacterial contamination. All steps were performed under sterile conditions within a laminar flow hood. Yeasts were placed in 2 mL cryovials containing 25 porous beads in cryopreservative broth (Deltalab, Rubí, Spain) and stored at −20 °C until use.

To recover yeast cells after beer fermentation, each beer sample was mixed to ensure homogenisation by inverting the bottle several times; then, 50 mL was transferred to a conical tube and centrifuged at 1845× *g* for 20 min at 4 °C to collect cells. Pellets were stored at −20 °C until use.

### 2.2. DNA Extraction

After beer fermentation, genomic DNA was isolated from axenic cultures and cells using a Thermo Scientific Yeast DNA Extraction Kit (Jena, Germany).

For axenic cultures, fresh yeast cultures were obtained by inoculating 1% (*v*/*v*) of cryopreservative broth stock on YPD agar and YPD broth (25 mL in sterile 50 mL conical tubes) at 25 °C for 3 days on an orbital shaker (S100D Optic Ivymen System, Madrid, Spain) at 200 rpm. To ensure the absence of extracellular bacteria before DNA extraction, yeast cultures were repeatedly grown in YPD medium with chloramphenicol, and their purity was checked by observing yeast cells under a phase-contrast microscope (NIKON ECLIPSE 50i, Nikon Europe B.V., Amstelveen, The Netherlands). Subsequently, yeast cells were harvested via centrifugation at 665× *g* for 5 min (Centrifuge 4k15, SIGMA, Rödermark, Germany) and resuspended in 200 µL of kit extraction buffer [31].

Cell pellets from beer fermentation were resuspended with 1 mL TE buffer (Tris 10 mM, EDTA 1 mM, and pH 8.0) and transferred to 1.5 mL tubes. Samples were centrifuged at 7380× *g* for 10 min at 4 °C, and pellets were resuspended in 200 µL of kit extraction buffer [32].

Borosilicate glass beads (diameter: 0.5 mm) were then added to samples, and yeast cells were lysed using a Digital Disruptor Genie (Scientific Industries, Inc., New York, NY, USA) for 4 min at full speed, with intermittent pauses in which the tubes were placed on ice to prevent potential overheating. Genomic DNA was extracted using the abovementioned kit, following the manufacturer’s protocol. DNA quality and quantity were measured using a 2000 series Nanodrop (Thermo Fisher, Waltham, MA, USA) and a Qubit 4 fluorometer (Thermo Fisher), respectively. DNA concentrations were reduced to 20 ng/μL working stock for polymerase chain reaction (PCR) amplification, and samples were stored at − 20 °C until further use.

### 2.3. Amplification and Sequencing of 16S rRNA Gene

To determine the presence of bacterial communities in the samples, the amplification and sequencing of the V3–V4 region of the 16S rRNA gene was performed using the primers 341F (CCTACGGGNGGCWGCAG) and 805R (GACTACHVGGGTATCTAATCC) [33], as this primer pair has considerable taxonomy coverage in bacteria and archaea [34]. For sequencing, the primers were ligated with Illumina overhang adapter sequences at the 5′ end (forward tail (341F) 5′-ACACTCTTTCCCTACACGACGCTCTTCCGATCT; reverse tail (805R) 5′-GACTGGAGTTCAGACGTGTGCTCTTCCGATCT).

PCR was performed in triplicate. The PCR mix contained 0.25 μM of each primer, 40 ng of template DNA, 25 μL of Supreme NZYTaq II 2× Colourless Master Mix (MB359, NZYTech, Lisboa, Portugal), and nuclease-free water (AppliChem GmbH, Darmstadt, Germany) at a volume of up to 50 μL. For controls, samples without yeast cells were processed using the DNA extraction protocol. Amplification was conducted using the SimpliAmp™ Thermal Cycler (Applied Biosystems, Thermo Fisher Scientific, Madrid, Spain), and the PCR conditions included initial denaturation at 95 °C for 5 min, followed by 35 cycles at 94 °C for 30 s, 55 °C for 30 s, 72 °C for 1 min, and a final extension stage at 72 °C for 10 min. PCR products were detected and analysed via agarose gel electrophoresis (0.7–1.2%, *w*/*v*) using GreenSafe Premium (MB132, NZYTech, Portugal). Amplicons were purified using the NucleoSpin Gel and PCR Clean-Up Kit (Macherey-Nagel, Düren, Germany) and sequenced on an Illumina platform by Macrogen (Madrid, Spain).

### 2.4. Bacterial 16S rRNA Gene Analysis

Paired-end reads obtained from Illumina sequencing were merged using the Fast Length Adjustment of Short Reads 1.2.11 program (FLASH) [35]. The sequences were then trimmed, filtered, and clustered using the CD-HIT-OTU software (v.0.0.1, Illumina rRNA data) [36]. Short reads were filtered through this process, and long sequences were trimmed. Using CD-HIT-DUP [37], filtered sequences were clustered using a 97% identity threshold and assigned to operational taxonomic units (OTUs). In addition, chimeras were filtered out, and extra-long tails were trimmed [38]. Summary statistics were obtained using a feature table to ensure successful processing. OTUs with a relative abundance of ˂0.01% were filtered out. For taxonomic assignment, a Naïve Bayes classifier was trained on reads from the V3–V4 region, extracted from Greengenes2 backbone sequences [39].

Statistical analyses were performed using QIIME2 v 2023.2 [40] and R version 4.2.3 (phyloseq, vegan, patchwork, agricolae, FSA, rcompanion, ggplot2, and tidyverse packages).

Variations in the compositions of the bacterial communities among different types of samples (yeasts obtained from *Vitis* and *Quercus* hosts and yeasts from axenic cultures and fermentation samples) were analysed. Alpha diversity was estimated using phylogenetic (Faith’s phylogenetic diversity) and non-phylogenetic (Shannon index, Pielou’s evenness, Chao1 index, and observed species) methods. Differences in community composition (β-diversity) [41] were computed using the Bray–Curtis dissimilarity metric and tested via permutational multivariate analysis of variance (PERMANOVA) [42]. Variations in community composition among the samples were visualised using principal coordinate analysis (PCoA) based on the Bray–Curtis distance. Analysis of Compositions of Microbiomes with Bias Correction (ANCOM-BC) was used to test the statistical significance of differential taxon abundances between sample groups (*p* < 0.001) at levels 2 and 6. Graphical representations were created using the R software, QIIME, and Microsoft Excel 2.1.

## 3. Results

### 3.1. Confirmation of Axenic Yeast Cultures

The main objective of this study was to identify possible bacterial communities that could be hidden in wild yeasts; therefore, the initial step was to ensure that the starting yeast cultures were axenic. To confirm this, all yeast strains were examined via microscopy using viability stains after repeated subcultures in a YPD medium with chloramphenicol. After confirming that the yeast cultures were axenic, we examined the bacterial populations present to determine whether beer fermentation affected the possible bacterial populations. As culture-dependent methods tend to mask and underestimate the diversity of bacterial communities, 16S rRNA gene metabarcoding was performed in axenic yeast cultures, as well as in cells recovered from the same yeasts after the fermentation of a brewing wort.

### 3.2. Alpha Diversity Metrics

**Figure 1:**
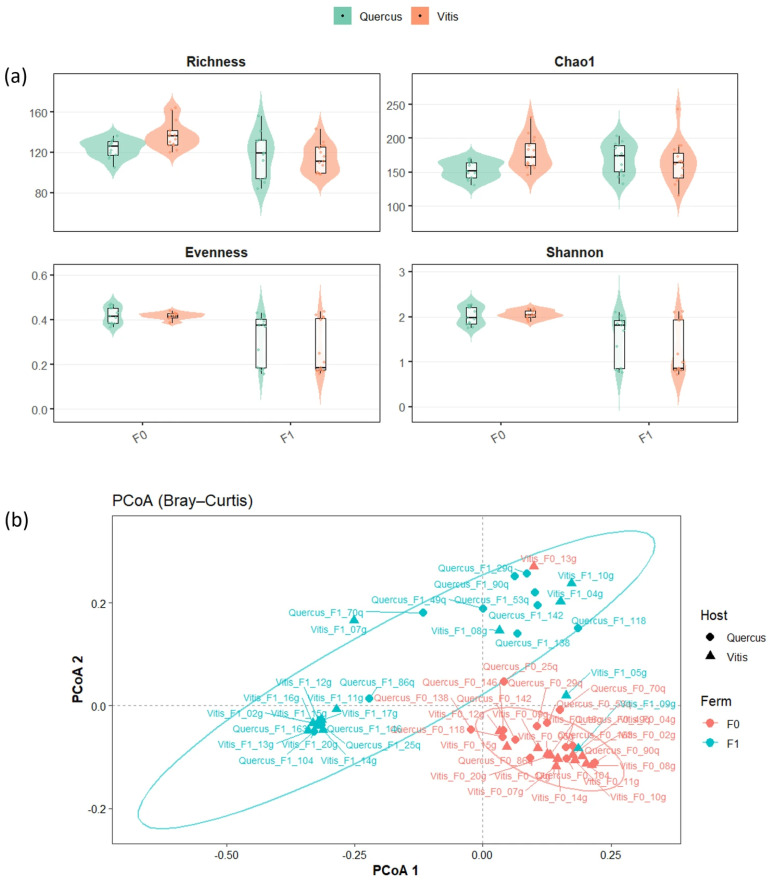
(**a**) Alpha diversity metrics for the bacterial communities: observed OTUs, Chao1 index, Pielou’s evenness, Shannon’s index. Data are represented as box plots, where the lower and upper parts of the box represent the first and third quartiles, respectively; the tick in the box indicates the median; and the ends of the whiskers indicate the minimum and maximum values. (**b**) Beta diversity of the bacterial communities, visualised through principal coordinate analysis (PCoA) based on the Bray–Curtis dissimilarity metric. In all cases, bacterial communities were analysed according to two factors: the yeast host (*Quercus* or *Vitis*) and the type of sample (axenic culture (F0) or fermentation sample (F1)).

Alpha diversity metrics (Faith’s phylogenetic diversity, observed OTUs, Shannon’s index, Pielou’s evenness, and Chao1 index) were calculated considering two factors: the yeast host (*Quercus* and *Vitis*) and the sample type (axenic cultures [F0] and fermentation samples [F1]). The results showed that there were significant differences in the bacterial communities detected in F0 and F1 samples regarding nearly all estimated alpha diversity metrics (Figure 1a–d and Table 1). Pairwise comparisons applied to F0 and F1 samples confirmed the significant differences (*p* < 0.05) in microbiome diversity between pure yeast cultures and cultures obtained after the fermentation process. No significant differences were detected between samples from *Quercus* ecosystems and those from *Vitis* plantations.

### 3.3. Beta Diversity Metrics

The beta diversity of the bacterial communities, visualised using PCoA based on the Bray–Curtis dissimilarity index, revealed some degree of clustering among data points according to the sample type (fermentation process [F1] vs. axenic yeast culture [without fermentation process, F0]) (Figure 1e). Moreover, the pairwise PERMANOVA test (Bray–Curtis distance) (999 permutations) confirmed that the bacterial community structure in the yeast varied significantly after fermentation in comparison with that seen in the axenic cultures (*p* < 0.001). No significant variation was observed between samples depending on their origin (*Quercus* vs. *Vitis*, *p* = 0.107) (Table 1).

**Figure 2:**
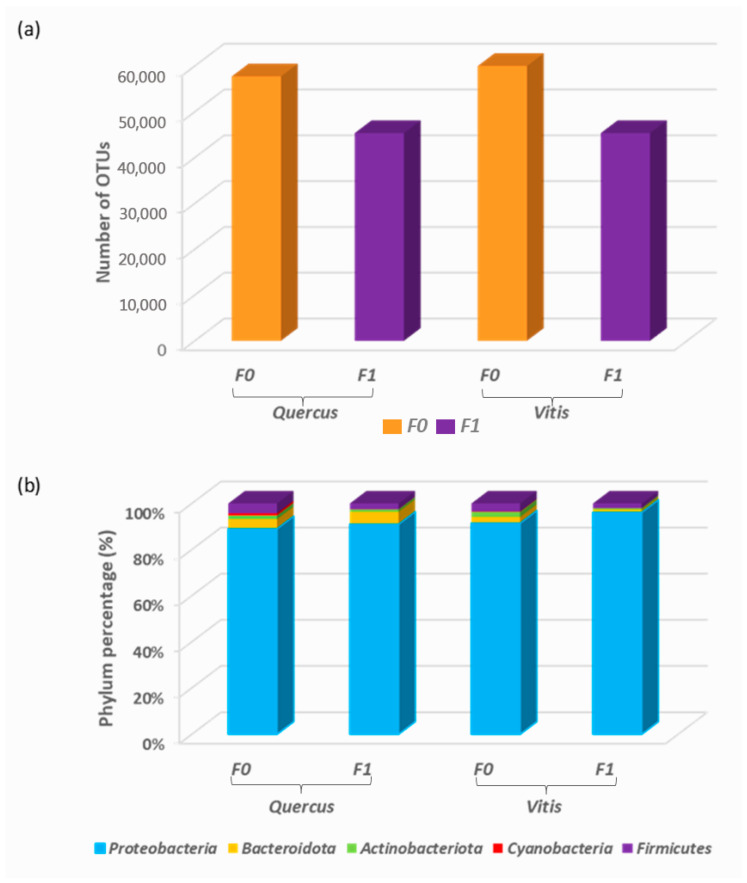
Numbers of operational taxonomic units (OTUs) (**a**) and relative composition of the bacterial community at the phylum level (**b**) considering both the sample type (axenic cultures (F0) and fermentation samples (F1)) and the yeast origin (*Quercus* and *Vitis* ecosystems).

**Figure 3:**
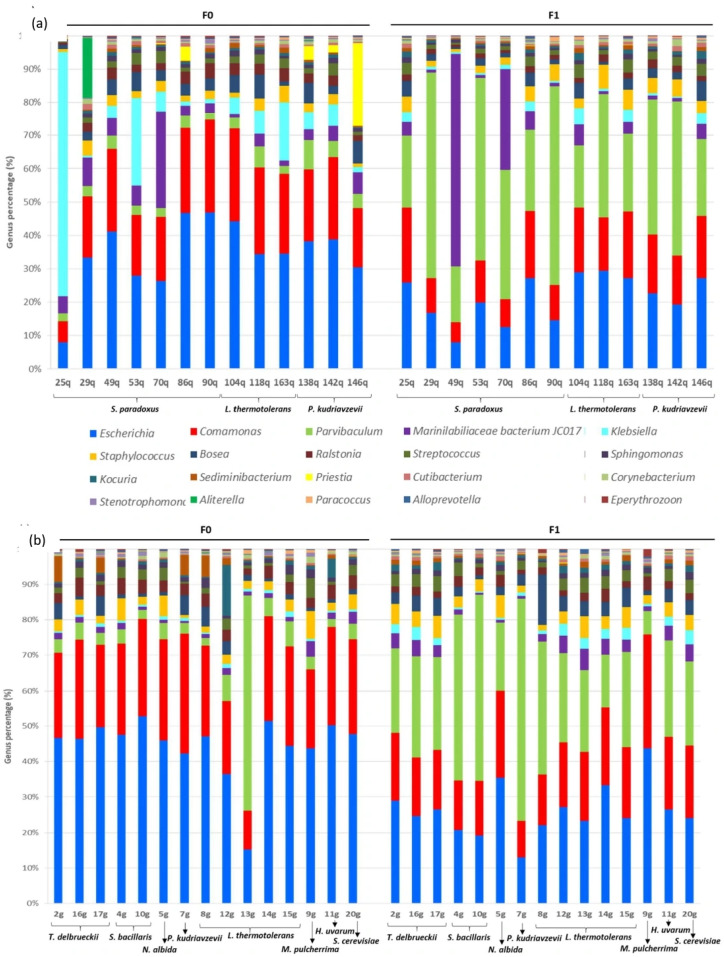
Relative composition of the bacterial community at the genus level, considering the sample type (axenic cultures (F0) and fermentation samples (F1)). Data are represented according the yeast origin: (**a**) *Quercus* and (**b**) *Vitis*.

Subsequently, the relative compositions of the bacterial communities were analysed at the phylum and genus levels (Figure 2 and Figure 3), and the data confirmed the similarities among *Quercus* and *Vitis* samples and the dissimilarities between samples from axenic yeast cultures and those from yeasts after wort fermentation, particularly at the genus level (Figure 3). Notably, the number of observed OTUs was higher in F0 than in F1 (Figure 2a). In most samples, the number of detected OTUs was significantly reduced from axenic cultures to fermented samples. For example, in yeast 25q, 6359 OTUs were found in F0 and 1445 in F1; moreover, in yeast 13g, 4780 OTUs were detected in F0 and 1124 in F1 (Supplementary Figure S1). However, some samples were observed to contain more OTUs after fermentation, especially among *Quercus* samples (Supplementary Figure S1). Among the different phyla (Figure 2b), Proteobacteria was the most abundant phylum in all studied strains, representing 92% of the detected OTUs. Other reads mapping to Bacteroidota (3.1%), Firmicutes (2.9%), Actinobacteria (1.4%), and Cyanobacteria (0.3%) were common among all yeasts (Figure 2b). In general, the relative abundance did not differ significantly between samples from *Vitis* and *Quercus* ecosystems or between axenic culture samples and those obtained post-fermentation. However, yeasts isolated from *Vitis* after fermentation exhibited the highest percentages of Proteobacteria, reaching more than 96% of the total reads, and almost no Actinobacteria were detected (0.05%) (Figure 2b).

Among the different genera (Figure 3), Escherichia (35% in *Quercus* and 44% in Vi-tis samples) and Comamonas (21% in *Quercus* and 25% in *Vitis* samples) were the predominant bacterial genera in F0 across both *Quercus* and *Vitis* yeasts. However, some exceptions were observed in which other bacterial genera constituted the majority, such as Klebsiella with 71% in the 25q strain or Parvibaculum with 58% in the 13g strain. In addition, in some strains, other bacterial genera represented more than 10% of reads, such as Kocuria in the 12g strain (13%), Aliterella in the 29q strain (16%), Priestia in the 146q strain (24%), and Marinilabiliaceae bacterium JC017 in the 70q strain (28%). In contrast, in F1, the bacterial populations present in yeasts of both origins (*Quercus* and *Vitis*) behaved similarly, with decreased percentages of Escherichia (20% in *Quercus* and 25% in *Vitis* samples) and Comamonas (14 in *Quercus* and 18% in *Vitis* samples) and increased percentages of Parvibaculum (*Quercus* from 3% to 34% in *Quercus* samples and *Vitis* from 3% to 29% in *Vitis* samples). Nonetheless, there were some exceptions, such as 49q, where a significant increase in reads for Marinilabiliaceae bacterium JC017 (59%) was observed. Two strains did not exhibit this evolution in bacterial genera after fermentation: the 13g strain, which initially had a very high percentage of Parvibaculum in F0, exhibited a decreased percentage of this genus after fermentation; moreover, the 9g strain, belonging to M. pulcherrima, showed no alteration in its bacterial composition during axenic culture due to the fermentation process. A possible explanation for this finding is that strain 13g does not provide favourable conditions for Parvibaculum growth during fermentation, possibly due to limited substrates, inhibitory byproducts, or competition with better-adapted microbes. In contrast, the community associated with strain 9g may be inherently stable or functionally redundant, meaning that fermentation did not exert sufficient selective pressure to alter its composition. These results suggest that the effects of fermentation may be strain-specific, depending on the initial community structure and microbial interactions.

The yeast species analysed showed similar profiles in terms of the percentage presence of endobacteria regardless of the yeast species—at least in the genera with the highest percentage values. However, exceptions included the axenic cultures of strains 25q and 13g of the *L. thermotolerans* species, where the presence of the genera *Klebsiella* and *Parvibaculum* was prominent. Nonetheless, after fermentation, these apparent differences were resolved in both strains, showing homogeneous profiles with regard to the other strains (Figure 3).

### 3.4. Differential Abundance

**Figure 4:**
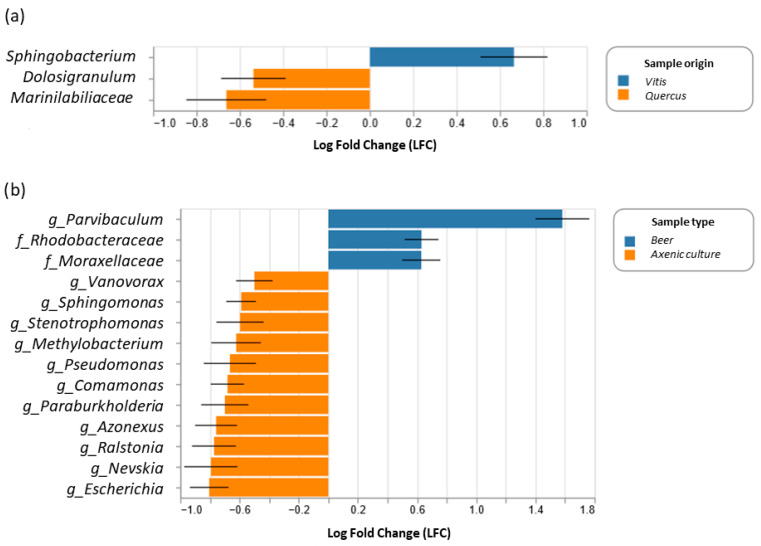
Differentially abundant bacterial taxa determined via Analysis of Compositions of Microbiomes with Bias Correction (ANCOM-BC). Only taxa satisfying a significant linear discriminant analysis are shown. Enriched and depleted bacterial genera between the two conditions are represented via blue and orange bars, respectively. (**a**) Comparison between different yeast origins (*Vitis* vs. *Quercus*) and (**b**) comparison between different types of samples (axenic cultures (F0) vs. fermentation samples (F1)).

The differentially abundant bacterial taxa among the different samples were derived using ANCOM-BC, comparing the bacterial genera found in yeasts from *Vitis* to those from *Quercus* (Figure 4a) and comparing those between yeasts in F0 and F1 (Figure 4b). This procedure allows the estimation of unknown sampling fractions and the correction of biases induced by sample differences. Absolute abundance data were modelled using a linear regression framework, providing a statistically valid test with appropriate *p*-values and confidence intervals for the differential abundance of each taxon and enabling control of the false discovery rate [43].

*Sphingobacterium* was more abundant in yeasts from *Vitis*, whereas *Dolosigranulum* and Marinilabiliaceae were more abundant in yeasts from *Quercus* (Figure 4a). In contrast, the genus *Parvibaculum* and the families Rhodobacteraceae and Moraxellaceae were more abundant in beer samples, but genera such as *Escherichia*, *Nevskia*, and *Ralstonia* were less abundant in samples collected from beer than in axenic cultures (Figure 4b).

## 4. Discussion

Endosymbiotic theory posits that a host cell engulfs bacteria and, instead of digesting them as food, establishes a functional relationship that enables the host cell/endosymbiont to utilise different components in energy metabolism [44]. Endosymbiotic bacteria play important roles in the evolution and diversification of eukaryotes [44]. The natural endosymbiotic association between yeasts and bacteria has become a major area of study, especially regarding biotechnological processes involving these organisms. Metabarcoding methods have several advantages over conventional methods used to study endosymbiotic associations, such as PCR, microscopy, and culture-based screening. Metabarcoding is particularly useful in investigating organisms that are difficult to isolate and detect using conventional methods. Moreover, culture-dependent methods tend to underestimate bacterial communities’ diversity.

The aim of this study was not to understand the microbial communities associated with wild yeasts but to provide evidence of bacterial communities in wild yeasts used in brewing applications that persist through the axenic culture process; it also sought to determine whether these communities were affected by the conditions inherent in fermentation. Previous studies have demonstrated the presence of hidden bacterial communities in yeasts [5,6]. Bacteria use yeasts as niches to survive under stressful conditions; therefore, yeasts can act as temporary or permanent bacterial reservoirs [6]. Notably, our study demonstrates that yeast strains and endobacterial communities develop together, and, depending on environmental factors, the bacterial community present within the yeast can be modified to contribute positively or negatively to the final product.

When studying wild yeast strains grown in the laboratory, it is important to consider the potential impacts of this process on the bacterial communities associated with such yeasts. For example, this process might alter the compositions of these bacterial communities and cause grouping between the bacterial communities of several types of yeasts, regardless of whether they originate from, e.g., *Quercus* or *Vitis*. Moreover, the fermentation process involves additional selection in these bacterial communities, promoting organisms that thrive under such conditions and restricting those that are less tolerant to fermentation-induced stress. In this study, the most abundant bacterial genera in axenic cultures were *Escherichia* and *Comamonas*, which are both Gram-negative bacteria. *Escherichia* belongs to the family Enterobacteriaceae, or enteric bacteria, with the best-known species being *E. coli.* Enteric bacteria are a largely homogeneous group within Proteobacteria; its members are all facultative aerobes, and most inhabit the intestinal tracts of humans or animals. *E. coli* is a model organism in the study of bacterial metabolism; it is relatively robust and grows well in various defined laboratory media and over a wide range of temperatures, normally in the presence of oxygen, although it can also grow under anaerobic conditions (as a facultative anaerobe) [45]. Therefore, its increased proportions in axenic yeast cultures may have been influenced by the growth conditions of the laboratory. Furthermore, in a recent study, auxotrophic strains of *E. coli* were used to generate synthetic *E. coli*–yeast endosymbionts, showing that the endosymbiosis between the two organisms allowed the bacterium to survive in the yeast cytosol using the cofactors/amino acids that it lacked from the host (*S. cerevisiae*) and supplying ATP to the yeast [46]. *Comamonas* belongs to the family Comamonadaceae, with 24 characterised species, and it grows well on routine bacteriological media [47]. Its members are common environmental bacteria associated with environmental bioremediation, and they occasionally cause human disease. Their natural habitats include soil, wastewater/sludge, fresh water, and animal intestinal tracts [48]. *Comamonas* is strictly aerobic, motile, non-pigmented, oxidase- and catalase-positive, and non-spore-forming, and it has a non-fermentative chemoorganotrophic metabolism [49,50].

After fermentation, the predominant changes observed in the bacterial composition were a decrease in the genera *Escherichia* and *Comamonas* and an increase in the genus *Parvibaculum. Parvibaculum* is a member of the Rhodobiaceae family and comprises four described species. The best-known species in this genus is *Parvibaculum lavamentivorans*. This is a mesophile that tolerates NaCl concentrations of 0–3% and can grow using acetate, ethanol, pyruvate, succinate, alkanes (C8–C16), and various anionic and non-ionic surfactants as carbon sources; however, it cannot grow on sugars. In a study of the complete genome of *P. lavamentivorans* DS-1T, no valid candidate genes were predicted for sugar or amino acid/peptide uptake systems [51,52]. Moreover, it grows very slowly on complex media such as LB or peptone agar plates. These characteristics could explain why this genus was poorly detected in axenic cultures in the present study; nonetheless, its abundance increased significantly after fermentation.

Finally, our findings were supported by the alpha and beta diversity indices, as the endobacterial community analysis revealed that the sample origin and the use of fermentation vs. non-fermentation significantly influenced the alpha and beta diversity values. Although the two sets exhibited largely shared genera, the % abundances of certain genera differed between the hosts and fermentation processes. The coexistence of endobacterial communities in laboratory cultures and in brewing experiments under laboratory conditions suggests that axenic cultures of wild yeasts may serve as niches or reservoirs for endobacterial communities, whose diversity is affected by biotechnological processes. Notably, the yeast appeared healthy when observed under a microscope, suggesting that the bacteria present were not pathogenic or virulent. These results could lead to new approaches to the application of wild yeasts in brewing and biotechnological processes.

The identification of endogenous bacterial communities associated with wild yeasts has direct practical implications for both brewing processes and biological control applications. Studies of spontaneously fermented beers and other mixed fermentation systems have shown that bacteria can coexist with yeast species, contributing to the complexity of microbial consortia and influencing flavour and fermentation dynamics [53,54].

In the context of brewing, the findings highlight that fermentation initiated with wild or non-conventional yeasts may not be microbiologically simple, even when the cultures are considered axenic. The presence of associated bacteria and diverse yeast taxa can influence the fermentation kinetics, sugar utilisation, stress tolerance, and the production of aroma-active metabolites, thereby affecting the flavour, stability, and reproducibility of beer. Monitoring such mixed microbial communities with high-throughput sequencing and other molecular tools could improve quality control, lead to more predictable fermentation outcomes, and enable brewers to intentionally select or design yeast–bacterium consortia to achieve the desired sensory profiles or functional properties [53,55].

In regard to biological control, the close associations between wild yeasts and their endogenous bacteria suggest that these microorganisms act as cooperative units rather than independent agents. Research on yeast–bacterium interactions has demonstrated that metabolic exchanges and ecological relationships can influence microbial survival, competition, and antagonistic activity, implying potential synergistic effects regarding pathogen suppression and colonisation efficiency [7,56]. Recognising and characterising these microbial partnerships would provide a strong basis for the development of more effective and robust biocontrol formulations that exploit naturally occurring microbial consortia, rather than relying on single strains.

## 5. Conclusions

This study reveals the presence of endobacterial communities in yeasts isolated from *Quercus* and *Vitis* under axenic conditions, showing that these yeast–bacterium associations primarily change in response to cultivation and fermentation conditions, rather than the ecological origin of the isolates. The results suggest that associated bacteria may play a role in brewing and biocontrol processes, although their obligate or transient nature and the factors influencing their persistence remain unclear. These findings underscore the need for standardised methods to detect, characterise and evaluate endobacterial communities. They also support a shift in perspective, from viewing wild yeasts as isolated production organisms to recognizing them as part of structured microbial consortia. This approach could improve process optimisation, product development and sustainable biotechnological applications, while future mechanistic studies are needed to clarify the functional basis of yeast–endobacterium interactions.

## Figures and Tables

**Table 1:** Summary of the alpha and beta diversity indices. In all cases, bacterial communities were analysed according two factors, Host and Ferm, and their interaction, Host*Ferm. Host refers to the yeast host, i.e., *Quercus* or *Vitis*, and Ferm refers to the type of sample, i.e., axenic culture (F0) or fermentation sample (F1).

	**Phylogenetic**	**Faith’s Phylogenetic Diversity**	**Factor 1**	**Factor 2**	**H Test ^a^**	***p*** **-Value**
			*Quercus*	*Vitis*	0.245	0.620
			F0	F1	1.803	<0.001 ***
	**Non-Phylogenetic**	**Chao1 Index**	**Df ^b^**	**Sum_sq ^c^**	**F ^d^**	**Pr (>F)**
		**Host**	1.0	2328.62	3.68	0.059
		**Ferm**	1.0	860	1.36	0.247
		Host*Ferm	1.0	1.491	2.35	0.129
		**Shannon’s Index**	**Df**	**Sum_sq**	**F**	**Pr (>F)**
		**Host**	1.0	0.25	0.80	0.372
		**Ferm**	1.0	13.21	42.42	<0.001 ***
**α-Diversity**		Host*Ferm	1.0	0.92	2.97	0.088
		**Pielou’s Evenness**	**Df**	**Sum_sq**	**F**	**Pr (>F)**
		**Host**	1.0	0.02	1.06	0.306
		**Ferm**	1.0	0.84	36.12	<0.001
		Host*Ferm	1.0	0.08	3.49	0.065
		**Observed OTUs**	**Df**	**Sum_sq**	**F**	**Pr (>F)**
		**Host**	1	2328.62	1.69	0.198
		**Ferm**	1	860	14.76	<0.01 **
		Host*Ferm	1	1.491	4.23	0.06
**β-Diversity**		**Bray–Curtis Dissimilarity**	**Df**	**Sum_sq**	**F**	**Pr (>F)**
		**Ferm**	1	1.27	22.58.	<0.001 ***
		**Host**	1	0.10	1.92	0.107
		**Residuals**	71	4.06	NaN	NaN
		**Total**	73	5.40	NaN	NaN

^a^ H test refers to Kruskal–Wallis test; ^b^ Df: degrees of freedom; ^c^ sum_sq: sum of squares; ^d^ F-distribution refers to Fisher–Snedecor distribution. Asterisks indicate significant differences: ** *p*-value < 0.01, and *** *p*-value < 0.001.

## Data Availability

The original contributions presented in this study are included in the article/Supplementary Material. Further inquiries can be directed to the corresponding authors.

## Supplementary Materials

The following supporting information can be downloaded at https://www.mdpi.com/article/10.3390/foods15020262/s1. Supplementary Figure S1. Evolution of the number of operational taxonomic units (OTUs) in yeasts isolated from *Quercus* (a) and *Vitis* (b), taking into account whether the sample came from an axenic culture (F0) or the fermentation process (F1). Supplementary Table S1. Information on the origin of the isolates analyzed in this study.
