# Supplementary material for: Wild Yeasts as Reservoirs of Bacterial Diversity: Biotechnological Insights from 16S rRNA Metabarcoding
Source: Foods. 2026 Jan 11;15(2):262. doi: 10.3390/foods15020262 (PMC12840430; doi:10.3390/foods15020262)
Supplement: Supplementary file 1 [file foods-15-00262-s001.zip › foods-4064923-supplementary.pdf]

SUPPLEMENTARY MATERIAL

Supplementary Table S1. Information on the origin of the isolates analyzed in this study.

Yeast species	ID Code	LOCATION	Sample	*HOST*
Hanseniaspora uvarum	11g	RIOJA	Grapes	*Vitis vinifera*
Lachancea thermotolerans	104q	ESKALMENDI	Leave	*Quercus faginea*
	118q	ESKALMENDI	Acorn	*Quercus robur*
	12g	RIOJA	Grapes	*Vitis vinifera*
	13g	RIOJA	Grapes	*Vitis vinifera*
	14g	RIOJA	Grapes	*Vitis vinifera*
	15g	RIOJA	Grapes	*Vitis vinífera*
	163q	IZKI	Bark	*Quercus pirenaica*
	16g	RIOJA	Grapes	*Vitis vinífera*
	8g	RIOJA	Grapes	*Vitis vinífera*
Metschnikowia pulcherrima	9g	RIOJA	Grapes	*Vitis vinífera*
Naganishia albida	5g	RIOJA	Grapes	*Vitis vinífera*
Pichia kudriavzevii	138q	ESKALMENDI	Acorn	*Quercus robur*
	142q	ESKALMENDI	Bark	*Quercus ilex*
	146q	ESKALMENDI	Soil	*Quercus robur*
	7g	RIOJA	Grapes	*Vitis vinífera*
Saccharomyces cerevisiae	20g	RIOJA	Grapes	*Vitis vinífera*
Saccharomyces paradoxus	25q	ULLIVARRI ARRAZUA	Soil	*Quercus faginea*
	29q	ULLIVARRI ARRAZUA	Leave	*Quercus faginea*
	49q	IZARRA	Leave	*Quercus robur*
	53q	IZARRA	Leave	*Quercus robur*
	70q	LANDA	Bark	*Quercus robur*
	86q	SALINAS DE LENIZ	Bark	*Quercus petraea*
	90q	SALINAS DE LENIZ	Soil	*Quercus petraea*
Starmerella bacillaris	10g	RIOJA	Grapes	*Vitis vinífera*
	4g	RIOJA	Grapes	*Vitis vinífera*
Torulaspora delbrueckii	17g	RIOJA	Grapes	*Vitis vinífera*
	2g	RIOJA	Grapes	*Vitis vinífera*

a)

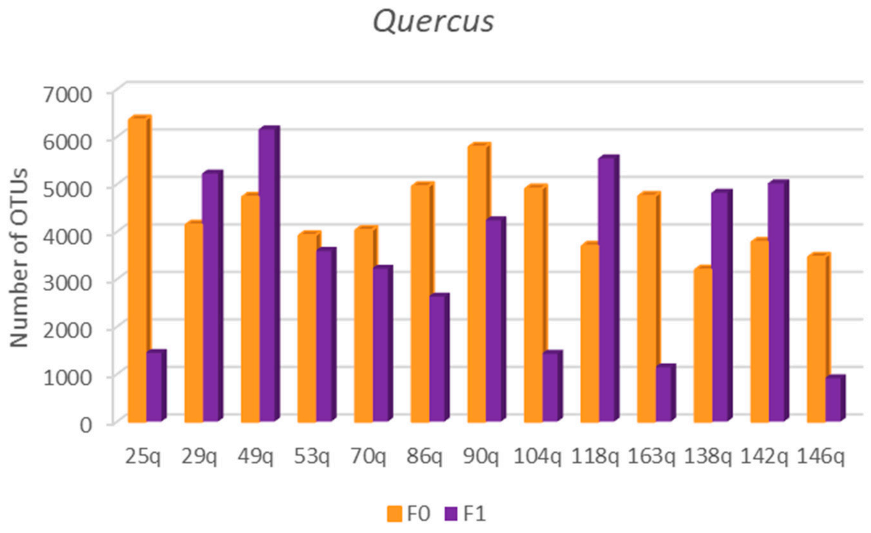

b)

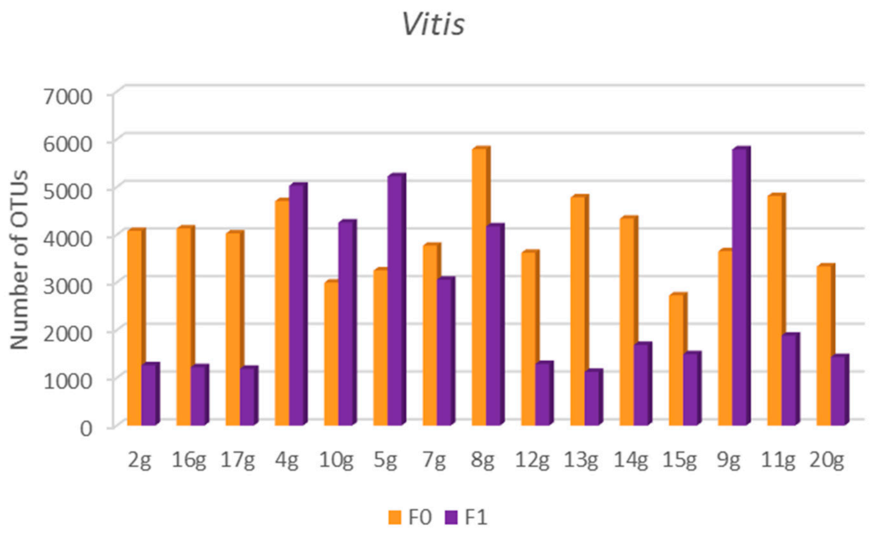

Supplementary Figure S1. Evolution of the number of Operational Taxonomic Units (OTUs) in yeasts isolated from *Quercus* (a) and *Vitis* (b) taking into account whether the sample came from axenic culture (F0) or from the fermentation process (F1).
